# Influenza and RSV incidence during COVID-19 pandemic—an observational study from in-hospital point-of-care testing

**DOI:** 10.1007/s00430-021-00720-7

**Published:** 2021-10-04

**Authors:** Paul Stamm, Ingo Sagoschen, Kerstin Weise, Bodo Plachter, Thomas Münzel, Tommaso Gori, Markus Vosseler

**Affiliations:** 1 Department of Cardiology, Cardiology I, University Medical Center Mainz, Geb. 605, Langenbeckstr. 1, 55131 Mainz, Germany; 2 German Center for Cardiovascular Research (DZHK), Partner Site Rhine-Main, Mainz, Germany; 3 Institute for Virology, University Medical Center Mainz, Mainz, Germany

**Keywords:** Point-of-care testing, Polymerase chain reaction, SARS-CoV-2, COVID-19 influenza, RSV, In-hospital, Prevalence

## Abstract

The severe acute respiratory syndrome coronavirus 2 (SARS-CoV-2) pandemic has forced the implementation of unprecedented public health measures strategies which might also have a significant impact on the spreading of other viral pathogens such as influenza and Respiratory Syncytial Virus (RSV) . The present study compares the incidences of the most relevant respiratory viruses before and during the SARS-CoV-2 pandemic in emergency room patients. We analyzed the results of in total 14,946 polymerase chain reaction point-of-care tests (POCT-PCR) for Influenza A, Influenza B, RSV and SARS-CoV-2 in an adult and a pediatric emergency room between December 1, 2018 and March 31, 2021. Despite a fivefold increase in the number of tests performed, the positivity rate for Influenza A dropped from 19.32% (165 positives of 854 tests in 2018/19), 14.57% (149 positives of 1023 in 2019–20) to 0% (0 positives of 4915 tests) in 2020/21. In analogy, the positivity rate for Influenza B and RSV dropped from 0.35 to 1.47%, respectively, 10.65–21.08% to 0% for both in 2020/21. The positivity rate for SARS-CoV2 reached 9.74% (110 of 1129 tests performed) during the so-called second wave in December 2020. Compared to the two previous years, seasonal influenza and RSV incidence was eliminated during the COVID-19 pandemic. Corona-related measures and human behavior patterns could lead to a significant decline or even complete suppression of other respiratory viruses such as influenza and RSV.

## Introduction

Since the outbreak in Wuhan in December 2019, the severe acute respiratory syndrome coronavirus 2 (SARS-CoV-2) has spread around the world and caused more than 4 million deaths [1, 2]. A number of different measures were implemented by governments around the world to curb the mortality increase observed in the countries that were hit first [3–5]. With the help of different public health care measures, such as isolation, mouth-and-nose protection, physical distancing, contact tracing, high-frequency testing, disinfection, etc., SARS-CoV-2 transmission could partially be controlled. Notably, these same interventions also have an immense influence on other infectious diseases, including influenza and Respiratory Syncytial Virus (RSV) [6–9].

Nucleic acid-based methods like polymerase chain reaction (PCR) testing represents the gold standard for detection of viral infections [10]. More recently, so-called point-of-care testing (POCT) has been developed to allow accurate and quick diagnosis of viral infections, and thus a more effective allocation and isolation of patients and timely application of antiviral therapy [11]. The combination of POCT and PCR (POCT-PCR) testing represents a complex and expensive test procedure, but with its accuracy and speed it has clear advantages especially in a highly frequented emergency rooms [12, 13]. Due to its relevance, single or combined POCT-PCR tests for SARS-CoV-2 have been developed and are increasingly used in acute care.

Here, we analyzed the POCT-PCR data in our internal medicine (adults) and general children’s emergency room from December 2018 until March 2021. We report on the incidences of viral respiratory diseases in direct comparison before and since the COVID-19 pandemic. In addition to the well-known SARS-CoV-2 infection and mortality rates, we particularly wanted to show the development of the seasonal spread of influenza and RSV recently. These insights should also allow conclusions to be drawn about the effects of corona-related mitigation strategies for similar viral pathogens.

## Methods

In a single-center retrospective observational study, we analyzed anonymized data from patients who received emergency treatment in the adult and children’s emergency department at the University Medical Center Mainz, a tertiary referral hospital in Germany (Rhineland-Palatinate), from December 1, 2018 to March 31, 2021. We report on the results of in total 14,946 consecutive tests for viral respiratory diseases performed by nasopharyngeal swab sample and assessed by POCT–PCR. Since the study involved an anonymized, retrospective analysis of diagnostic standard data, ethics approval was not required according to German law.

**Fig. 1:**
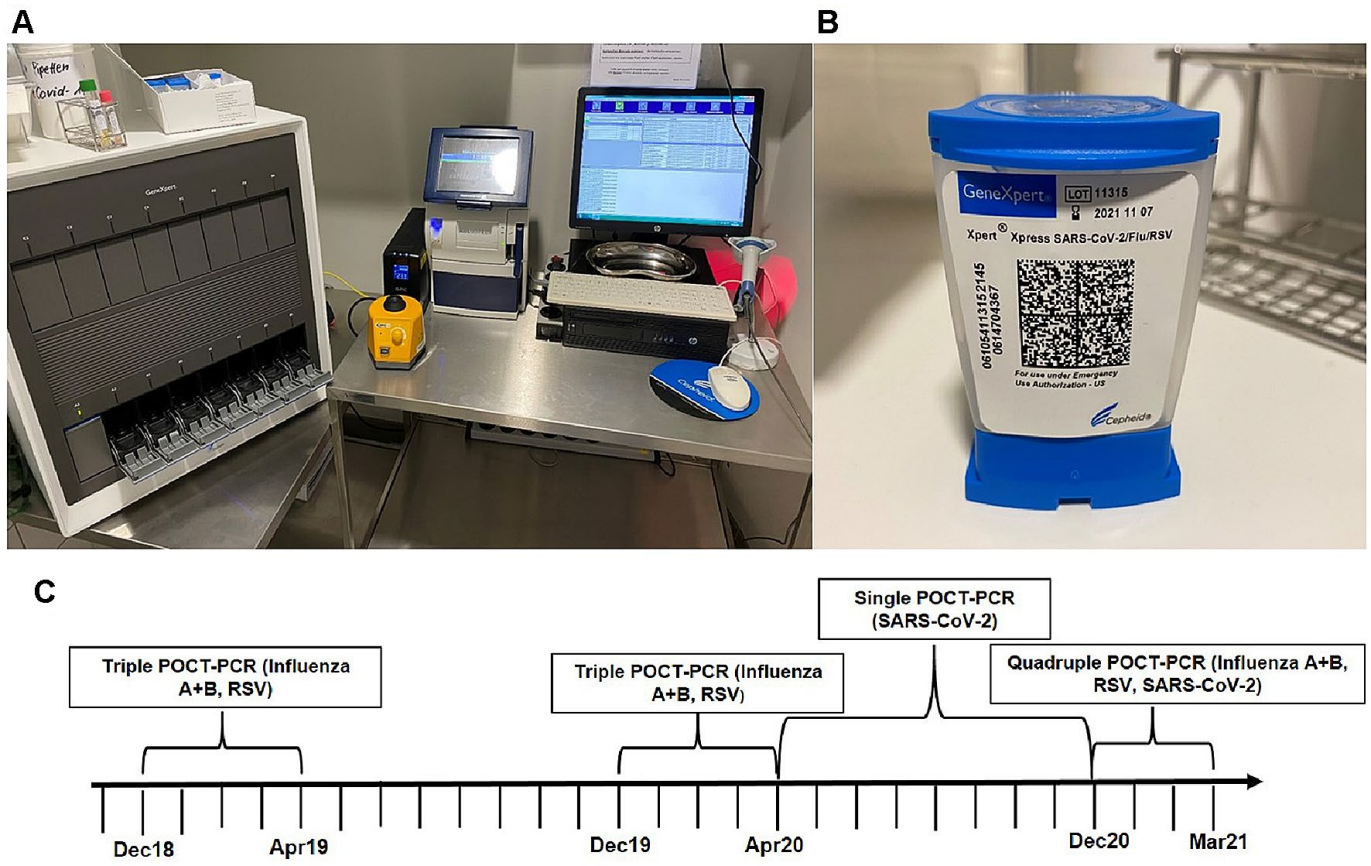
Instrument, cartridge and scheme of the POCT-PCR testing. **A** POCT-PCR workstation with GeneXpert XVI-16 module instrument (left) and GeneXpert Xpress Software on a desktop computer (right). **B** Xpert Xpress Cartridge for Quadruple-POCT-PCR test. **C** Schematic representation of the respective test strategy from December 2018 to March 2021

Depending on the season or the viral occurrence an interdisciplinary conference (virologists, internists, pediatricians and hygiene doctors) decided which viruses to test for in the respective period. During the influenza seasons 2018/2019 and 2019/2020, a Triple-POCT (Influenza A, Influenza B, RSV) was performed. Within the SARS-CoV-2 pandemic, a single SARS-CoV-2-POCT was used from April 2020 until November 2020. From December 2020 to March 2021, a Quadruple-POCT (Influenza A, Influenza B, RSV, SARS-CoV-2) was performed (Fig. 1C). In inter-seasonal periods (May–November 2019 and 2020), no POCT–PCR testing was performed. The switch to the quadruple test took place immediately as the corresponding cartridge was commercially available. Due to the limited availability, the inclusion criteria for POCT testing changed over time. In the beginning of the pandemic, only symptomatic patients with a possible SARS-CoV-2 Infection were tested. In late 2020, as the pandemic proceeded and more test cartridges became available, all patients who presented in the emergency room and were scheduled for inpatient treatment in our hospital were tested.

POCT-PCR testing was performed using Cepheid GeneXpert System (Sunnyvale, CA, USA). GeneXpert XVI-16 module instrument was accessed by the GeneXpert Xpress Software on a desktop computer (Fig. 1A). The exact test procedure can be seen on the manufacturer’s manual [14]. Briefly, the swap was transferred via pipette in a Xpert Xpress Cartridge (Fig. 1B). The PCR method was performed after the cartridge was loaded into the GeneXpert instrument. The official virological report was then validated by the colleagues from Institute of Virology.

## Results

**Fig. 2:**
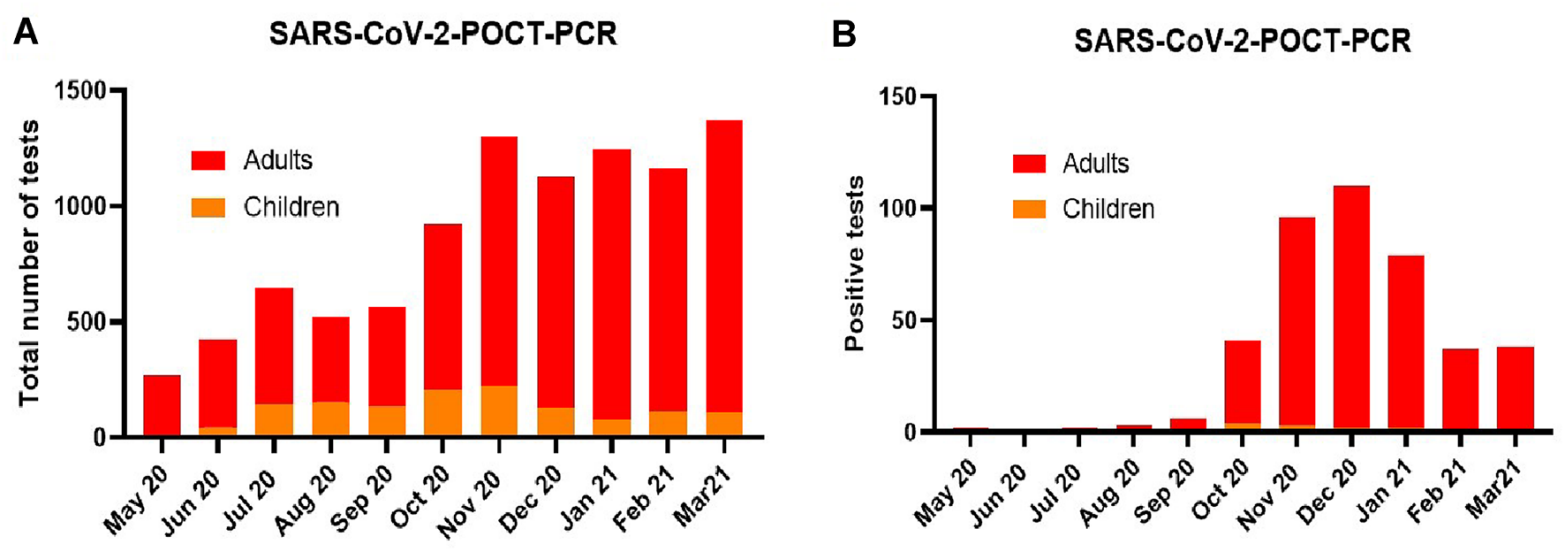
POCT-PCR tests for SARS-COV-2 from May 2020 to February 2021. **A** The number of POCT-PCR tests for SARS-COV-2 performed monthly increased until November 2020 and then stayed stable around 1200 total tests per month. **B** During the so-called second SARS-CoV-2 wave, the positive test rate increased from September 2020 and reached its maximum in December 2020 with a subsequent decrease

From May 1, 2020 until March 31, 2021 a total of 9557 POCT-PCR test for SARS-COV-2 were performed in our emergency rooms - 1337 of them in our children’s emergency room. The test capacity was expanded from 300 tests in May 2020 to over 1000 monthly tests since November 2020 (Fig. 2A) corresponding to the beginning of the influenza season.

The infection rates in summer 2020 were very low resulting in few positive tests in our emergency units (Fig. 2B). During the so-called “second coronavirus wave” from autumn 2020, an increase in the positivity rates with a peak of 110 (9.74%) positive tests in December 2020 was observed. Since then, following the implementation of further containment measures, the infection rate dropped to 2.77% (38 of 1373) positive tests in March 2021. Overall, we registered a very low positivity rate of 0.97% (13 positives of 1337 tests) for SARS-CoV-2 infections in children.

**Fig. 3:**
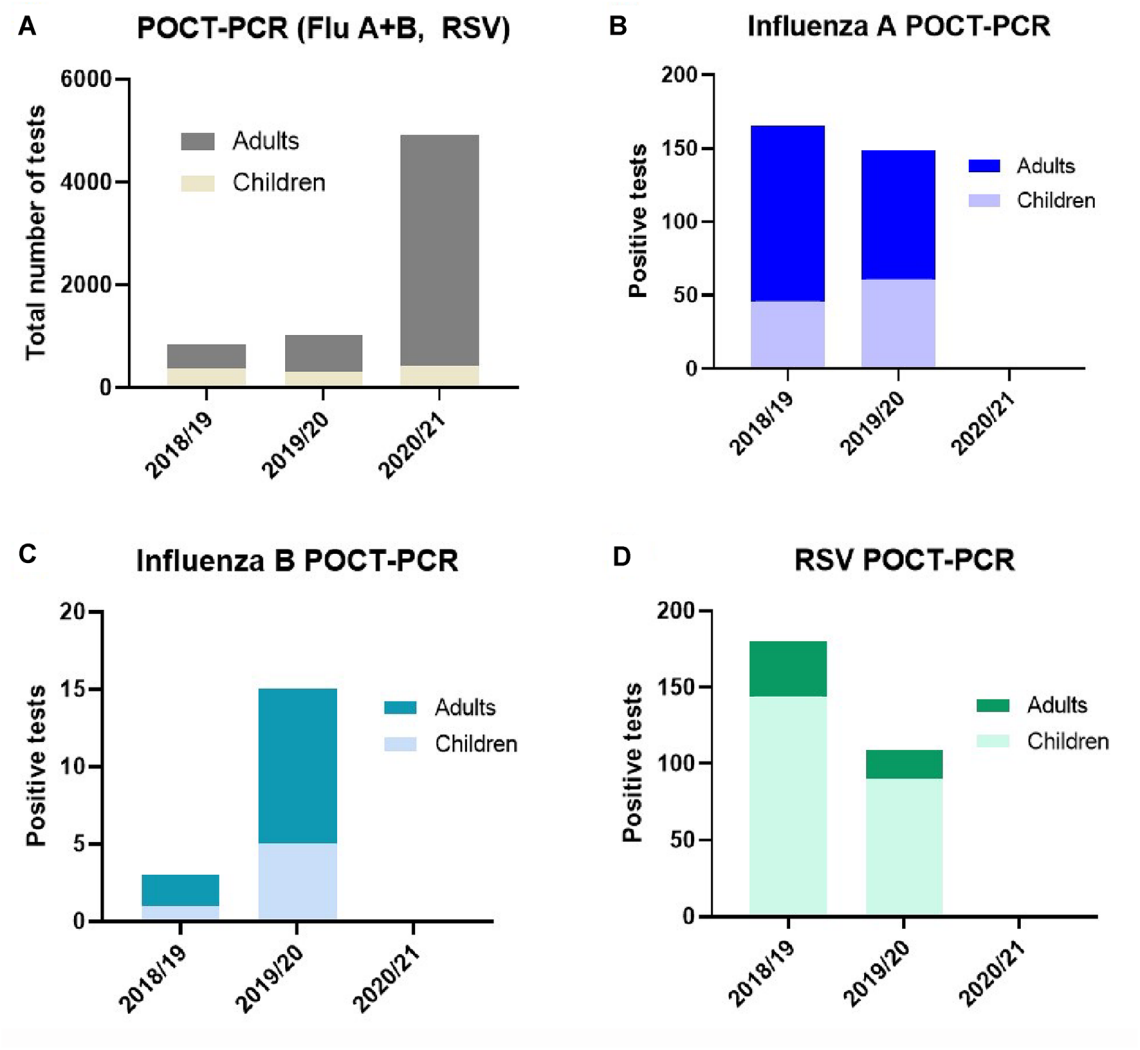
POCT-PCR tests for Influenza A, Influenza B and RSV from 2018 to 2021. **A** The total number of seasonal POCT-PCR tests for Influenza A, Influenza B and RSV was roughly the same in the 2018/19 and 2019/20 seasons and increased by fivefold in 2020/21. **B**–**D** The number of positive tests for Influenza and RSV in the 2018/19 and 2019/20 seasons completely disappeared in 2020/21

The number of tests for Influenza A, Influenza B and RSV (Fig. 3A) included in the analysis increased by about fivefold between 2018/19 and 2019/20 winter/spring seasons and 2020/21 after the implementation of the Quadruple-POCT. Despite this fivefold increase in the number of tests performed, the positivity rate for influenza A dropped from 19.32% (165 positives of 854 tests in 2018/19), 14.57% (149 positives of 1023 in 2019–20) to 0% (0 positives of 4915 tests) in 2020/21 (Fig. 3B). Similarly, none of the tests were positive for Influenza B or RSV in the 2020/21 season until the end of March 2021 (Fig. 3C, D). A similar number of Triple-POCT tests were performed in our pediatric emergency room over the three consecutive seasons. It should be noted that children were primarily responsible for RSV infections (approx. 90%) and accounted for about 30% of the influenza infections in 2018/19 and 2019/20.

**Fig. 4:**
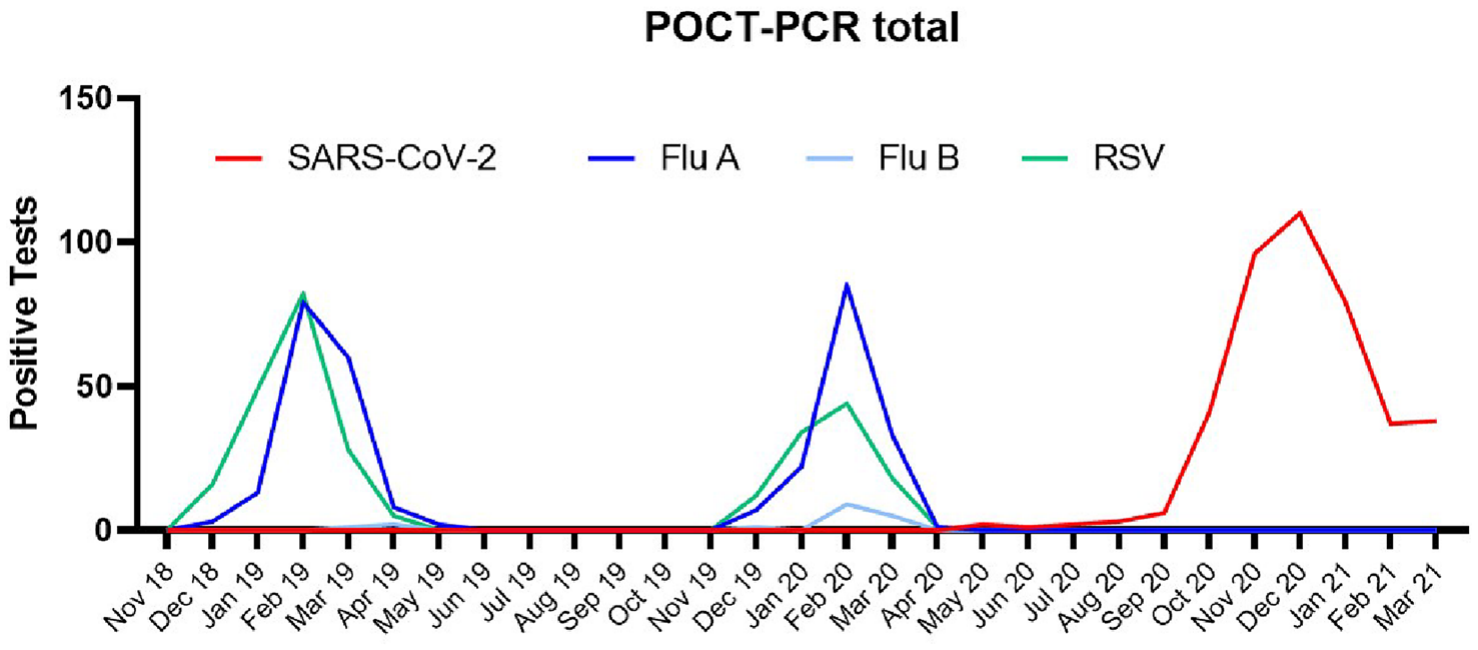
Overall positive POCT-PCR tests 2018–2021. During the COVID-19 pandemic, Influenza and RSV infections disappeared when compared to previous seasons

In the 2018/19 and 2019/20 seasons, we were able to determine a relevant incidence of infections with Influenza A and RSV using the POCT-PCR method in our emergency units (Fig. 4). February in particular turned out to be the annual peak of these viral respiratory infections in our region. Since the COVID-19 pandemic and establishment of a POCT-PCR-based detection method (first Single- then Quadruple-POCT) in our adult’s and children’s emergency rooms, we did not observe any infection with Influenza A, B or RSV until the end of March 2021.

## Discussion

Social distancing, mouth-and-nose protection, disinfection and frequent hand-washing represent basic measures to protect against SARS-CoV-2, influenza and RSV according to the World Health Organization advices [15]. Due to the danger posed by SARS-CoV-2 and resulting COVID-19, these rules have become much more relevance in the general public and have also been implemented in state regulations and laws [16]. We speculate that these strict public health measures and individual-level hygiene precautions against COVID-19 resulted in a dramatic decline of influenza and RSV incidence in Germany and worldwide.

Despite an increase in the number of tests performed and no change in the indication to perform these tests, as compared to the previous two years, the SARS-CoV-2 pandemic was associated with the disappearance of influenza and RSV infections. Although we only analyzed POCT-PCR data from emergency rooms of a single hospital, these correspond to the general incidence rates for influenza and SARS-CoV-2 in Germany (according to the Robert Koch Institute) and Europe [1, 17, 18].

Even more than a year after the outbreak of SARS-CoV-2 in Wuhan, there is no really convincing pharmacological therapy for COVID-19—apart from minor exceptions [19–21]. For this reason, in addition to large-scale vaccination programs, the public health measures still have an important role in reducing the spread of the virus [22, 23]. Due to the appearance of virus variants and a possible attenuation of SARS-CoV-2 vaccine protection over time, lifestyle changes and lockdowns remain effective strategies to control the virus.

However, these strategies not only have a direct impact on SARS-CoV-2 itself, but also they have relevant ancillary effects. While some of these include depression, isolation and limited access to health care, they also lead to a decreased spread of several other viruses, including those that show a recurrent typical seasonal dynamic. Similarly, a marked decrease in measles infections was registered in 2020 after the number of infections had increased in previous years [24–26]. There was also a decrease in varicella and rubella infections due to COVID-19 control measures [27]. An analogous decrease of sexually transmitted diseases and food-borne diseases was also observed [28]. In Switzerland, a substantial reduction in almost all recorded infectious diseases in 2020 was observed when compared to previous years [29]. All in all, the observed decline in these infectious diseases could be mainly attributed to the COVID-19 control strategies. Nevertheless, due to the alignment of the health system towards SARS-CoV-2, an under-reporting or misdiagnosis could also have played a role.

Other research groups have already published similar results: Lee et al. showed that the national response strategies in Korea not only reduced SARS-CoV-2 cases, but also substantially decreased influenza activity when compared with recent seasons [30]. Chan et al. and Sun et al. came to similar conclusions in the Northern Hemisphere and China [31, 32].

In a recent article, there is a similar reduction in influenza transmission in the European region for the 2020/21 season as in the present study [33].

The higher reproduction number (R0) of SARS-CoV-2 in comparison with other respiratory viruses like influenza or RSV could explain the big differences in the impact of the public health measures on viral circulation and disease incidence [34].

## Conclusions

The medium- and long-term consequences of the COVID-19 pandemic are still largely unclear. The medical and economic impact of SARS-CoV-2 is not limited to the effect of the virus, but also impacts on the prevalence of other infections with analogous transmission. Compared to the two previous years, seasonal influenza and RSV incidence was eliminated during the COVID-19 pandemic. In direct comparison, however, SARS-CoV-2 appears to be clearly superior to other respiratory viruses in terms of resistance and infectivity which is why effective preventive and therapeutic strategies continue to appear indispensable.

## Limitations

The current study has some limitations. POCT-PCR tests for Influenza A, Influenza B, RSV and SARS-CoV-2 were analyzed in a monocentric retrospective observational design. Since we only used POCT-PCR, there were no clearly defined test criteria; the primary implementation was dependent on an experienced triage nurse (MTS) and different test settings were used due to the seasonality of the viruses, so our study could be limited by selection bias. The comparable low infection rates with Influenza B could be caused by regional or demographic distribution.
